# Understanding Delayed Diabetes Diagnosis: An Agent-Based Model of Health-Seeking Behavior

**DOI:** 10.1177/0272989X251326908

**Published:** 2025-04-04

**Authors:** Firouzeh Rosa Taghikhah, Araz Jabbari, Kevin C. Desouza, Arunima Malik, Hadi A. Khorshidi

**Affiliations:** Business School, University of Sydney, NSW, Australia; Faculty of Business Administration, Université Laval, QC, Canada; Faculty of Business and Law, Queensland University of Technology, QLD, Australia; School of Physics, University of Sydney, Camperdown, NSW, Australia; Melbourne School of Population and Global Health, The University of Melbourne, Melbourne, VIC, Australia; School of Computing and Information Systems, The University of Melbourne, VIC, Australia

**Keywords:** decision analytics, decision support systems, health behavior, noncommunicable disease, complex systems simulation

## Abstract

**Background:**

Diabetes is a rapidly growing global health issue, with the hidden burden of undiagnosed cases leading to severe complications and escalating health care costs.

**Methods:**

This study investigated the potential of integrated behavioral frameworks to predict health-seeking behaviors and improve diabetes diagnosis timelines through the development of an agent-based model. Focusing on Narromine and Gilgandra in New South Wales, Australia, the model captured the integrative influence of 3 social theories—theory of planned behavior (TPB), health belief model (HBM), and goal framing theory (GFT)—on health care decisions across behavioral and nonbehavioral variables, providing a robust analysis of temporal diagnostic patterns, health care utilization, and costs.

**Results:**

Our comparative experiments indicated that this multitheory framework improved predictive accuracy by 15% to 30% compared with single-theory models, effectively capturing the interplay of planned, belief-driven, and context-based health behaviors. Spatial-temporal analysis highlighted key regional and demographic variations in diagnosis behaviors. While early, planned medical visits were prevalent in regions with better access (Gilgandra), areas with limited infrastructure saw a reliance on hospital-based diagnoses (Narromine). Health care cost analysis demonstrated a nonlinear expenditure pattern, suggesting that these theories defy conventional linear cost trends. Scenario analysis demonstrated the impact of targeted interventions. Gender-specific awareness initiatives in Gilgandra reduced late-diagnosis rates among men by approximately 15%, while enhanced access to care in Narromine decreased hospital-based late diagnoses from a baseline of 80% to around 60%.

**Conclusions:**

This study contributes an empirically grounded, policy-oriented decision support tool to inform targeted interventions, offering novel insights to improve diabetes management.

**Highlights:**

- We explored the delay in diabetes diagnosis, particularly within remote Australian communities, through looking into the health care–seeking behavior of individuals displaying diabetes symptoms.
- We developed an innovative agent-based model to craft a dynamic decision support tool for policy makers by providing unique insights into the health behaviors of diabetes patients.
- Our study contributes significantly to the understanding of public health management with particular concerns around diabetes, as well as equips the New South Wales Ministry of Health with impactful insights into the consequences of their decisions.

## Graphical abstract

**Graphical Abstract:**
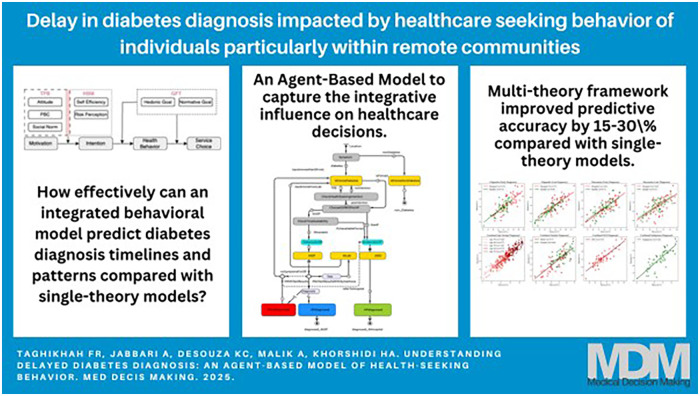
This is a visual representation of the abstract.

## Introduction

Diabetes represents one of the most urgent and rapidly escalating global health challenges of the 21st century.^1^ As the fastest-growing chronic condition worldwide, it stands among the leading causes of mortality.^1^ Projections indicate that by 2050, 1.3 billion people will be living with the condition.^2^ The economic impact is equally profound, as exemplified in Australia, where diabetes-related health care costs, government subsidies, and ongoing care exert a substantial financial burden of approximately AU$17.6 billion annually.^3^ These factors highlight the far-reaching consequences of diabetes, demonstrating the urgent need for comprehensive management strategies to curb its spread and mitigate associated health costs.

A critical and often overlooked aspect of diabetes is the high prevalence of undiagnosed cases. Current data reveal that, in Australia, for every 5 individuals diagnosed, 4 cases remain undetected.^4^ This hidden burden is exacerbated by the widespread tendency among individuals to disregard early symptoms, such as fatigue, frequent urination, and excessive thirst. Alarmingly, more than 40% of individuals with these symptoms delay seeking medical advice for more than a year, unaware of their potential link to diabetes.^5^ The delay in diagnosis and treatment significantly heightens the risk of multiple chronic conditions presenting simultaneously at initial diagnosis, escalating both health risks and health care costs. This delay is attributable not only to the disease’s gradual, asymptomatic progression—which can remain undetected for an average of 12 y before clinical diagnosis^6^—but also to behavioral factors.

Studies indicate that a significant portion of the population may not identify the symptoms of diabetes, underscoring a critical gap in health awareness and the behaviors necessary for timely diagnosis and care.^7^ This gap is particularly concerning given that early diagnosis in primary health care settings is essential for effective diabetes management and can help prevent the progression to more severe stages often seen in hospital diagnoses, where intervention opportunities may already be limited. Various behavioral theories seek to explain the complex factors contributing to health-seeking delays and diagnostic timelines, each offering insight into individual and social motivators.^8^ For instance, the health belief model (HBM)^9^ and self-regulation theory^10^ propose that individuals’ actions are largely influenced by their perceived threat of illness and readiness to pursue diagnosis.^11^ However, these theories often overlook the impact of social or cultural factors that influence perceptions of health and shape attitudes and beliefs about illness and medical care.^12^ Recognizing these broader influences is crucial for understanding the delays in health-seeking behavior and supporting interventions that promote early diagnosis within primary health care settings, where timely intervention can have the most substantial impact.

The transtheoretical model of change^13^ and protection motivation theory^14^ highlight how individuals assess health risks and their coping abilities and gradually become more prepared to take health-related actions. However, these theories tend to overlook the external resources, health care availability, or support needed to progress through stages. For instance, socioeconomic status (SES) often determines an individual’s access to quality health care and health information, affecting their ability to get a timely diagnosis.^15^ Another relevant framework is the theory of planned behavior (TPB), which emphasizes that an individual’s intention to engage in health-seeking behaviors is influenced by attitudes toward the behavior, subjective norms, and perceived behavioral control (PBC).^16^ TPB fails to account for the impact of the perceived risk of disease and the systemic barriers to accessing care.^17^ Thus, while the above theories provide valuable information, none offers a comprehensive view that encapsulates the complex interplay of social, economic, and individual factors affecting health-seeking behaviors in chronic disease management. This points to the need for a more integrative approach.

This study investigated the effectiveness of an integrated behavioral framework in predicting and analyzing diabetes-related health-seeking behaviors. Specifically, our study focused on the following 3 research questions (RQs):

I. How effectively can an integrated behavioral model predict diabetes diagnosis timelines and patterns compared with single-theory models?
II. How are health care costs, utilization rates, and diagnostic performance metrics different between hospital-based and medical center–based diabetes diagnosis under varying behavioral frameworks?
III. How do theory-based interventions influence late diagnosis rates in different demographic and health care settings?

To address these questions, we proposed a theoretical framework grounded in 3 carefully selected behavioral theories: TPB, HBM, and goal framing theory (GFT).^18^ These theories were chosen because each offers distinct yet complementary insights into the motivations behind health behaviors in diabetes care. TPB examines the planned and conscious aspects of decision making, while HBM focuses on how risk perception, severity, and barriers to care drive motivations. HBM also provides insights into why individuals delay seeking medical attention. GFT addresses how contextual goals shape individual choices, which are crucial for understanding the influence of convenience and social expectations on health care utilization.

To translate this multitheory framework into a dynamic and testable model, we employed agent-based modeling (ABM). This is a computational approach suited for simulating complex, multilevel interactions between agents and their environments. In this study, ABM bridged the gap between social sciences and medical research^19–21^ by allowing for a realistic simulation of individual decision-making processes^22–24^ and their collective impact on health care outcomes, such as diagnosis timelines and the preference for hospital-based versus primary care diagnoses. This approach has demonstrated its value in the investigation of communicable diseases, providing information on behaviors such as protective measures in influenza transmission^25^ and other infectious diseases.^26,27^ In the broader health care context, ABM has been successfully applied in areas such as selection of health care providers,^28^ reducing waiting times,^29^ and formulating health policies,^24,30^ underscoring its versatility in modeling diverse health care scenarios.

Narromine and Gilgandra, located in New South Wales (NSW), Australia, were selected as case study regions because of their unique characteristics that make them particularly suitable for examining behavioral influences on diabetes management and prevention. These regions face significant health care challenges, including high diabetes prevalence, limited health care access, and some of the highest rates of potentially preventable hospitalizations and avoidable deaths in NSW, resulting in substantial unnecessary health care costs. The regions also have significant Indigenous populations, who make up approximately 20% of the Narromine population and 14% of the Gilgandra population according to Australian Bureau of Statistics (ABS). This demographic feature is particularly relevant because Indigenous Australians experience diabetes rates nearly 3 times higher than non-Indigenous Australians, with diabetes being the second-leading cause of death for Aboriginal and Torres Strait Islander people in 2018.^31^ The complex health care challenges faced by these underserved communities necessitate a behavioral analysis approach to better understand and address the barriers to early diagnosis.

This study contributes to the field of diabetes management and health care delivery in 3 key ways. First, the comprehensive behavioral framework synthesizes multiple theoretical perspectives to capture the complex interplay of individual, social, and environmental factors that influence health-seeking behaviors. Second, the developed computational model uniquely captures the characteristic of real-world health care decisions. Unlike traditional simulation models that focus mainly on cost-effectiveness and screening strategies,^32,33^ our ABM incorporates bottom-up emergent dynamics and social interactions, providing a more realistic representation of health-seeking behaviors. Third, the ABM was developed through a participatory modeling process that improves its credibility and practical utility. This collaborative approach produces a trusted decision support tool that enables policy makers and health care providers to design and evaluate targeted interventions to improve early diagnosis rates and optimize resource allocation.

## Materials and Methods

We developed an agent-based computational model to investigate health-seeking behavior in individuals with diabetes, at both aggregated (macro) and individual (micro) levels. The agents’ actions were informed by human-like reasoning, modeled by personal subjective/perception of their environment, beliefs, preferences, and experiences. The method aptly encapsulates the macro-level patterns emerging from micro-level interactions, providing valuable semi-quantitative insights into collective behaviors and unveiling causal links behind system-level phenomena.^24,34^ This makes it suitable for studying the complexity of cumulative effects of scenarios targeting changes in individual behavior.

In developing this model, extensive stakeholder engagement enabled comprehensive validation and refinement of the behavioral dynamics. The collaborative process involved key health care and industry experts, including 5 members from the NSW Ministry of Health Data Science Group, who brought expertise in health policy, data analytics, and population health, and 4 specialists from Ernst & Young (EY) with backgrounds in health care consulting and systems modeling. Through a series of 8 structured workshops conducted over 4 mo, these experts provided critical input during the model development, validation, output analysis, and experiment design phases. Each workshop was systematically documented by the project manager and facilitator to capture valuable insights and recommendations. This rigorous engagement process strengthened the model’s credibility by validating key assumptions, refining decision-making rules, and ensuring the model accurately represented real-world health care–seeking behaviors in the context of diabetes management.

The technical implementation used the AnyLogic software platform for the agent-based simulation, complemented by Python-based empirical machine learning analyses. Our modeling approach strictly adhered to the Overview, Design Concepts, Details protocol,^35^ ensuring methodological transparency and reproducibility. Model development and validation involved iterative consultation with key stakeholders, including health care providers and policy makers, to ensure alignment with real-world dynamics.

### Overview

#### Purpose

##### What was the purpose of the model?

Our agent-based model investigated the complex interplay of behavioral and nonbehavioral factors that influence health-seeking patterns among individuals with undiagnosed diabetes and the general population.

#### Entities, state variables, and scales

##### What kind of entities were in the model?

The model incorporated 3 distinct agent types or entities that interacted within the health care ecosystem: patient agents represented individual health care consumers, medical health center agents modeled primary health care facilities, and hospital agents simulated tertiary care institutions.

##### By what state variables or attributes were these entities characterized?

Patient agents were characterized by multidimensional attributes, including sociodemographic factors such as age, gender, education, income, ethnicity; health status indicators such as disease progression, diabetes comorbidities, mental health, health behaviors, mental distress; and cognitive-normative factors such as health literacy, cultural health belief, barriers to health service, trust, perceived accessibility, continuity of medical health, community connections.

Health service agents (both medical centers and hospitals) were defined by operational characteristics such as cost structure, service availability, capacity; performance metrics such as precision of diagnosis, efficacy of treatment; and accessibility factors such as location, waiting times, service hours.

##### What were the temporal and spatial resolutions and extents of the model?

**Figure 1:**
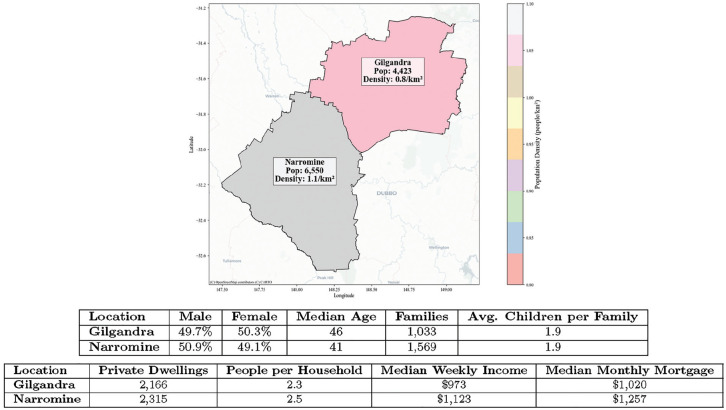
Gilgandra and Narromine SA2.^a^ ^a^The image shows the geographic location, while the tables provide demographic information for each area.

This study focused on the Gilgandra and Narromine Statistical Area 2 in the far west of NSW, Australia (refer to Figure 1), because of the higher rate of diabetes.^23^ According to the Australian Institute of Health and Welfare (AIHW), the age-standardized rate of type II diabetes hospitalizations in this region was significantly higher than the national average in 2015–2016 to 2017–2018.^31^ The temporal unit of the model was a day, reflecting daily self-care behavior, and the simulation was extended for approximately 11 y (4,000 d). By this time, all diabetes patients in the 2 study regions were projected to be diagnosed.

All agents operated within a geographic information system (GIS)–based environment, which provided a spatially realistic model of their surroundings, including health care facilities, residential areas, and distances between locations. The GIS framework was essential because it allowed the simulation to incorporate real-world geographic and spatial data, making it possible to analyze how factors such as distance to health care facilities and community layout influenced health-seeking behaviors.

#### Process overview

##### What entity did what, in what order?

In this simulation, agents regularly checked their health status, with the frequency of these checks varying based on their demographic characteristics and any symptoms they may be experiencing. Agents who had no health issues remained at home, requiring no further action. However, for those who identified symptoms during these routine checks, their decision to seek medical care was influenced by their health-seeking intentions. If they opted to pursue care, they could choose between visiting a hospital or a medical center. Hospital visits led directly to the emergency department, where a diagnosis was guaranteed on the first visit owing to a 100% diabetes diagnosis rate. Medical centers, however, did not ensure an immediate diagnosis. If symptoms were noticeable, medical centers could diagnose diabetes by referring patients for lab tests; however, if symptoms were mild, there was a probability that the physician may choose not to proceed with further screening and send the patient home, leaving them undiagnosed. This may lead to a delayed diagnosis, because patients may need multiple visits to receive proper testing. Agents with mild symptoms who returned repeatedly to the medical center might eventually receive a diagnosis, but if the center continually failed to identify diabetes, the patient might decide to seek care at a hospital. In urgent cases, medical centers referred patients directly to hospitals for immediate treatment. The limited operating hours of medical centers also influenced patient choices; if a center was closed or the waiting times were long when urgent care was needed, agents proceeded directly to the nearest hospital. After each visit, agents provided feedback on the quality of care they received, which shaped their future health care–seeking decisions. More information is available in Appendix A.1.

##### Was the order imposed or dynamic?

The sequence in which state variables were updated in the simulation was managed through both imposed and dynamic ordering. Imposed order referred to a structured, predetermined sequence that governed key decisions, such as when agents assessed their symptoms and decided whether to seek medical care. This ensured that decisions related to health-seeking behavior followed a consistent order across agents. Dynamic order, in contrast, adapted based on evolving interactions and circumstances, such as changes in an agent’s social network or their accumulated experience with health care services.

##### How was time modeled?

Time was modeled in a continuum over which continuous processes and discrete events could occur. In all the equations, the current time was represented as *t*, while *t*− 1 signified the time in the previous period. Thus, any changes or transitions occurring between these 2 time points reflected the modeled system’s dynamics over 1 step.

### Design Concepts

#### Basic principles

##### Which general approaches for concepts, theories, or hypotheses were included in the model’s design?

**Figure 2:**
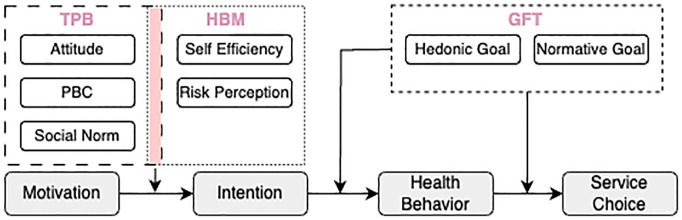
An overview of applied behavioral theories in agent-based modeling to explain health behavior and choice of service. In GFT, gain goal was excluded due to its irrelevance to the study context. GFT, goal framing theory; HBM, health belief model; TPB, theory of planned behavior.

We considered 3 behavioral theories—TPB,^16^ HBM,^9^ and GFT^18^—to understand the reasoning behind health-seeking behavior for a population of undiagnosed patients (see Figure 2). The selection of these theories over others was driven by their complementary perspectives in explaining health-related behavior. TPB provided insights into how social norms and PBC influenced health-seeking behavior, while HBM captured individual perceptions of disease threat and treatment benefits. GFT complemented these by explaining how different motivational frames—hedonic and normative—simultaneously affected decision making in health care contexts. These theories have demonstrated strong empirical validity across diverse health care settings^36^ and collectively addressed the cognitive, social, and motivational dimensions of health-seeking behavior and choice of health care service, making them particularly suitable for understanding diabetes management.

##### How were they taken into account?

TPB examined the relationship between an individual’s beliefs, abilities, perceptions, and actions. The 3 main components of TPB included attitude, PBC, and subjective norms.^34^ TPB posits that an individual’s intent to act is influenced by cognitive, control, and normative beliefs. This theory has been examined in several studies related to patients’ delay in seeking treatment for mental health issues,^37^ addictive behavior, oral problems, cancer,^38^ eating disorders, or chronic diseases.^39^ It has been used successfully to study the self-care and self-efficacy behavior of patients with prediabetes and develop diabetes prevention interventions.^40^ HBM complemented TPB by considering an individual’s perceived susceptibility to and severity of illness and their self-efficacy in following treatment recommendations.^41^ It incorporated the value that individuals placed on their health and treatment.^42^ GFT guided our understanding of the factors influencing agent choices in care providers. It suggests that individual decision-making processes are influenced by 3 overarching goals: hedonic (seeking immediate comfort), normative (following social norms), and gain (maximizing resources).^43^ In our study context, where health care services were provided free of charge through Australia’s universal health care system, gain goals related to financial considerations played a minimal role in decision making. Therefore, our model focused on hedonic and normative goals, which better captured the immediate comfort-seeking behaviors and social influences that drove health care service utilization in this setting.

#### Emergence

##### What key results emerged from the adaptive traits or behaviors of individuals?

This model explored the emergence of 2 interrelated phenomena: diabetes health-seeking behavior and the choice of health service. These behaviors emerged from individual interactions with health care providers and each other. The dynamic distribution of early and late diagnosed patients, as well as the frequency of health professional visits, was a result of individuals’ decisions based on perceived risks, costs, social influences, and evolving personal health conditions. The model incorporated stochastic elements to reflect real-world unpredictability in how individual characteristics and health service qualities changed over time. These results were tightly dependent on what agents did and were less imposed by the modeling rules.

#### Adaptation

##### What adaptive traits did the individuals have?

As diabetes progressed, the health status of the agents worsened, affecting their perceived benefits, costs, and risks of seeking health care. If the disease reached an advanced stage, agents were forced to seek emergency care, overriding their usual decision-making rules. Their perceptions of service quality, trust, and self-care were updated based on social influences and their previous interactions with health care providers.

#### Objectives

##### What was the main objective of agents as a group?

The primary objective for agents was to maximize their overall well-being through health-seeking behavior and choosing the appropriate health care provider. Agents used a utility-based decision model, incorporating factors such as attitude, PBC, social norms, risk aversion, and self-efficacy. The decision function balanced perceived benefits, costs, and risks, aligned with prospect theory, where losses were weighted more heavily than gains.

#### Learning

##### Did individuals change their behavior as a consequence of their experience?

The learning process for trust $TR_{i,j,t}$ in health care provider j by agent i was dynamic and evolved based on the outcome of each visit. Successful diagnoses reinforced trust, while unsuccessful diagnoses reduced it. This experience-based learning process adjusted dynamically with each interaction as in equation 1.

(1) $$TR_{i,j,t}=\alpha \cdot TR_{i,j,t-1}+\gamma \cdot DO_{i,j,t}-\delta \cdot \left(1-D_{i,j,t}\right)$$

where $TR_{i,j,t}$ is perceived trust in provider j at time t . $DO_{i,j,t}$ is the diagnosis outcome at time *t*, where $D_{i,j,t}=1$ if the diagnosis is successful and $D_{i,j,t}=0$ otherwise. δ is the penalty parameter that scales the reduction in trust when a diagnosis fails. α and γ are weight parameters, with α capturing persistence of past trust and γ adjusting trust increase for successful diagnoses.

The initial trust $TR_{i,j,0}$ was based on the health care provider’s reputation, which served as a starting point. Trust increased with each successful diagnosis, while failed diagnoses reduced trust, creating an adaptive learning process based on outcomes.

#### Sensing

##### What were agents assumed to know or perceive when making decisions?

Patient agents were assumed to know the health condition, lifestyle choices, and the choices of health service of themselves and other agents. Information was complete and certain. The sensing was local through social networks. The structure of the network was both imposed and emergent, and the mechanism through which agents obtained information was explicit in a process.

#### Interaction

##### What forms of interaction were there between agents?

Agents interacted within their social networks, sharing information about health behaviors and experiences with health care services. These interactions shaped social norms, which influenced agents’ decisions regarding health care–seeking behaviors. The strength of social influence in these interactions was based on behavioral consistency rather than fixed relationship parameters. This influence was dynamically updated as agents observed how consistently their contacts engaged in health-seeking behaviors. The influence of contact k on agent i at time t was modeled as in equation 2:

(2) $$IN_{i,k,t}=\frac{\sum_{\tau =t-\Delta t}^{t}I\left(a_{k,\tau }=HS_{i,t}\right)}{\Delta t}$$

where $I(a_{k,\tau }=HS_{i,t})$ is an indicator function returning 1 if contact k performed the same health behavior $HS_{i,t}$ at time τ and 0 otherwise. Δt is the defined time window over which consistency is measured, allowing $IN_{i,k,t}$ to reflect recent behavior patterns.

The reputation score $RP_{i,j,t}$ captured the collective satisfaction within the social network of agent i regarding health care provider j . Reputation evolved based on recent feedback from network contacts, with recent perceptions weighted more heavily as in equation 3.

(3) $$RP_{i,j,t}=\lambda \cdot RP_{i,j,t-1}+\left(1-\lambda \right)\cdot \frac{1}{\left|NU_{i}\right|}\sum_{k\in N_{i}}FE_{k,j,t}$$

where $RP_{i,j,t}$ is the reputation score of health care provider j for agent i at time t , based on network feedback. $N_{i}$ is the set of agents in the social network of agent i who provide feedback on provider j . $FE_{k,j,t}$ is the satisfaction or feedback score of agent k (a network contact of i ) on provider j at time t . λ is the decay parameter that emphasizes recent feedback (values closer to 1 give more weight to past reputation).

#### Stochasticity

##### What processes were modeled by assuming they were random or partly random?

Several processes in the model employed stochastic elements to capture real-world variability. These included an agent’s initial health status, the trust level in health care providers, the diagnosis rate at health centers, and appointment availability. Stochasticity was also applied to interactions within the social network, introducing randomness in how agents perceived social norms. This randomness reflected inherent uncertainties in health behaviors and provider interactions.

#### Observation

##### What data were collected from the ABM for testing, understanding, and analysis?

Data collected from the model included daily counts of undiagnosed and diagnosed diabetes patients, health care costs, patient distribution across services, and gender distribution in diagnosis rates. Statistical analyses, including regression models and machine learning techniques, were applied to identify patterns in health-seeking behavior, diagnosis timing, and health care service utilization. These insights helped pinpoint bottlenecks and the demographic effects of diabetes care.

### Model Details

#### Initialization

##### What was the initial state of the model world, that is, at time t=0 ?

The model initialized with a health function $H_{i,t=0}$ for each agent i , which represented their initial overall health status. This function incorporated chronic conditions that may affect diabetes progression and was normalized between 0 (completely healthy) and 1 (completely unhealthy). It is defined as in equation 4:

(4) $$H_{i,t}=\sum_{z}w_{z}C_{i,z}+p\cdot SI_{i,t}$$

where $C_{i,z}$ is the severity level of chronic condition z for agent i , normalized between 0 and 1. $w_{z}$ is the weight assigned to condition z , determined by expert input or other data. p is a scaling factor that modulates the influence of $SI_{i,t}$ on overall health. $SI_{i,t}$ is the diabetes severity index for agent i at time t , representing the ongoing impact of diabetes as defined in equation 5:

(5) $$SI_{i,t}=SI_{i,t-1}+r_{p}$$

where $r_{p}$ is the base rate of diabetes severity progression per month, which is different for diagnosed and undiagnosed diabetes.

##### How many entities of what type were there initially?

The simulation started with 4,101 patient agents in Gilgandra and 6,190 in Narromine, each with varying sociodemographic characteristics; 3 primary medical centers and 1 hospital in Gilgandra; and 1 medical center and 1 hospital in Narromine.

##### Was initialization always the same or was it varied?

To introduce variability and realism, the model included a random initialization function. This was achieved using a Monte Carlo technique that assigned initial states (e.g., health status, social ties) to agents based on random seed values. This ensured reproducibility while capturing the inherent randomness and variability in real-world population characteristics and behaviors.

##### Were the initial values chosen arbitrarily or based on available data?

The initial values were informed by diverse data sources, including epidemiological studies, demographic databases, and health surveys. For instance, demographic data from sources such as the ABS provided the initial distribution of agent ages, sexes, and other sociodemographic characteristics. Epidemiological studies shaped the progression rates of diabetes and the likelihood of developing complications. Behavioral data from health surveys guided patterns in physical activity, dietary habits, and access to health care services.

#### Input data

##### Did the model use input from external sources, such as data files or other models, to represent processes that changed over time?

The model used inputs as listed in Table 1.

**Table 1:** Input Data Sources for the Model Parameters.

Variable	Notation	Description	Data Source/Assumption
Health status	$HE_{i}$	Self-assessed health status	ABS National Health Survey
SES	$\mathrm{SE}S_{i}$	Comprehensive SES, including income, education level, and occupation	ABS Census Data
Health literacy	$HL_{i}$	Ability to understand and navigate health care systems	ABS Health Literacy Survey
Mental distress	$MD_{i}$	Psychological distress (depression, anxiety) that affects health decisions	ABS National Health Survey
Health behaviors	$HB_{i}$	Includes BMI, smoking, and alcohol consumption affecting health outcomes	AIHW Health Behaviors and ABS Health Survey
Cultural health beliefs	$CL_{i}$	Influence of cultural practices, particularly for Indigenous Australians	ABS Census and National Aboriginal and Torres Strait Islander Health Survey
Severity index	$SI_{i}$	A measure of health status specific to diabetes	AIHW Diabetes Data
Chronic condition	$C_{i,z}$	Existence of chronic conditions	ABS National Health Survey
Continuity of medical care	$E_{i,j,t}$	Consistency of care with health care providers over time	AIHW Health Community Indicators
Distance to health care	$\mathrm{DI}S_{i}$	Distance between individual and nearest health care provider (in km)	Google Maps and GIS environment
Max distance	MaxDIS	Maximum possible distance in the region	AIHW Rural Health Data
Travel time	$TT_{i,t}$	Time taken to travel to health care provider (in minutes)	Google Maps and GIS environment
Max travel time	TT	Maximum travel time in the region (minutes)	ABS Transport Data and GIS environment
Reputation	$RP_{i,j}$	Reputation of health care provider	Google Review
Trust	$TR_{i,j,t}$	Experience with and trust in health care providers	ABS Patient Experience
Barriers to health service	$BA_{i}$	Factors preventing use of health services when needed (e.g., time, cost, capacity)	ABS Patient Experience

ABS, Australian Bureau of Statistics; AIHW, Australian Institute of Health and Welfare; BMI, body mass index; GIS, geographic information system; SES, socioeconomic status.

### Submodels

#### What were the submodels that represented the processes?

As briefly mentioned in our process overview, our patient agent had 2 submodels, including health-seeking decision and choice of service.

#### What were the model parameters, their dimensions, and reference values?

The parameters of the submodels, along with descriptions of their values, are listed in Table 1. We defined age as a continuously changing parameter updated annually and also considered education changes proportionally for people between the ages of 20 and 40 y. Residential addresses were assigned to patient agents randomly within city borders. The impact of different diseases on agent i ’s health status was set to be equal at 0.5.

#### How were submodels designed or chosen, tested, and parameterized?

The following sections contain a complete description of the health-seeking decision function and choice of service function.

#### Health-seeking decision function

The health-seeking decision $HS_{i,t}$ for individual i at time t followed a utility-based framework, balancing perceived benefits, costs, and risks. We used a weighted utility model,^44^ in which agents evaluated gains and losses asymmetrically by integrating factors such as attitude, PBC, social norms, risk aversion, self-efficacy, and hedonic gains. This is represented in a compact form as in equation 6:

(6) $$HS_{i,t}=\frac{\sum_{k}\theta_{k}\cdot X_{i,k,t-1}}{\sum_{k}\theta_{k}}$$

where $X_{i,k,t-1}$ is each factor at time t−1 , including attitude $A_{i,t-1}$ , PBC $\mathrm{PB}C_{i,t-1}$ , social norms $SN_{i,t-1}$ , risk aversion $RA_{i,t-1}$ , self-efficacy, $SE_{i,t-1}$ , hedonic gains $HG_{i,j,t-1}$ . $\theta_{k}$ is the weight parameter for each corresponding factor $X_{i,k,t-1}$ , indicating its importance in the decision-making process, including $\theta_{A},\theta_{P},\theta_{N},\theta_{R},\theta_{S},\theta_{G}$ , respectively.

Agents with a score greater than or equal to 0.5 sought medical help. In severe cases, agents had to seek professional help immediately, regardless of their $HS_{i,t}$ score.

#### Attitude function

Attitude $A_{i,t}$ toward seeking health care for diabetes was influenced by various factors, including health status, SES, health literacy, mental distress, health behaviors, and cultural beliefs. Based on the utility model,^45^ a logistic function with a summation of weighted factors was used as in equation 7:

(7) $$A_{i,t}=\frac{1}{1+e^{-\sum_{k^{\prime }}\theta_{k^{\prime }}\cdot X_{i,k^{\prime },t}}}$$

where $X_{i,k,t}$ is each factor influencing attitude at time t , including $HE_{i,t}$ , health status at time *t* calculated in equation 4; $\mathrm{SE}S_{i}$ , SES; $HL_{i}$ , health literacy, crucial for understanding diabetes management; $MD_{i}$ , mental health status, such as not having depression or anxiety and other mental conditions, $HB_{i}$ , healthy lifestyle (checking for body mass index ( $\mathrm{BM}I_{i}$ ); smoking ( $SM_{i}$ ); alcohol consumption ( $DR_{i}$ )); and $CL_{i}$ , cultural health beliefs, particularly for Indigenous Australians. $\theta_{k^{\prime }}$ is the weight coefficient for each factor $X_{i,k,t}$ , representing the influence of each factor on the attitude function including $\theta_{h}$ , $\theta_{s}$ , $\theta_{h^{\prime }}$ , $\theta_{m}$ , $\theta_{b}$ , $\theta_{c}$ , respectively.

Research indicates that SES significantly affects health-seeking behavior, with lower socioeconomic levels correlated with reduced preventive health actions and increased delays in pursuing medical care.^46^ Furthermore, individuals with greater knowledge of disease symptoms tend to seek professional help more promptly. Enhanced health literacy plays a vital role, because it enables patients to better understand medical information and effectively navigate the health care system.^47^ Psychological factors, such as mental distress, can lead to avoidance behaviors and neglect of essential medical care, ultimately hindering diabetes management.^48^ Unhealthy lifestyle choices, including a high BMI, smoking, and alcohol use, are also associated with delayed treatment-seeking behavior, which can exacerbate diabetes complications and complicate disease management.^49^ Moreover, cultural beliefs shape individuals’ perceptions of illness and treatment, influencing their likelihood of seeking conventional medical care.^50^

#### PBC function

The PBC function $\mathrm{PB}C_{i,t}$ modeled an individual’s perceived ability to seek health care by incorporating both external barriers and self-efficacy. It is defined as in equation 8:

(8) $$\mathrm{PB}C_{i,t}=\theta_{p}\cdot \frac{1}{1+\log\left(1+BA_{i}\right)}+\theta_{e}\cdot SE_{i,t}$$

where $BA_{i}$ is the perceived barriers to health service use; $SE_{i,t}$ is self-efficacy, representing confidence in managing health at time *t*; and $\theta_{p}$ , $\theta_{e}$ are weight parameters for barriers and self-efficacy.

#### Social norms function

The social norms $SN_{i,t}$ reflected the influence of an individual’s social network on their health-seeking behavior, dynamically adjusting based on the frequency of interactions and behavioral alignment within the network. Studies indicate that lifestyles and health care behaviors are significantly affected by family, neighborhood, and community context.^51^ A network-based approach^52^ assigned influence weights that changed over time according to the level of engagement and shared behaviors as in equation 9.

(9) $$SN_{i,t}=\frac{1}{\left|NU_{i}\right|}\sum_{k\in N_{i}}\left(\frac{IN_{i,k,t}}{t}+\theta_{n}\cdot \frac{FQ_{i,k,t}}{FQ}\right)\cdot IN_{i,k,t}$$

where $NU_{i}$ is a set of social contacts for individual i and $FQ_{i,k,t}$ is the total frequency of interactions between i and k up to time t , normalized by FQ . $\theta_{n}$ is a weight parameter for the impact of interaction frequency on social influence and $IN_{i,k,t}$ is the influence exerted by contact k at time t on individual i as defined in equation 2. This function updated sequentially as the simulation advanced, ensuring that influence accumulated over time and reflected behavioral changes within the network.

#### Self-efficacy function

Self-efficacy $SE_{i,t}$ reflected the individual’s confidence in managing diabetes, based on previous experiences with health care providers. Consistent and positive health care experiences increased confidence, while negative experiences could reduce it.^53^ This function evolved based on success feedback, as in equation 10:

(10) $$SE_{i,t}=\theta_{e^{\prime }}\cdot SE_{i,t-1}+\theta_{j}\cdot \left(e^{EX_{i,j,t}}+e^{HE_{i,t}}\right)$$

where $EX_{i,j,t}$ is the continuity of care with health care provider j at time *t*, and $\theta_{e^{\prime }}$ and $\theta_{j}$ are the weight parameters for previous self-efficacy and for the influence of the exponential terms for health status and continuity of medical care.

#### Risk aversion function

The risk aversion $RA_{i,t}$ formulation was inspired by the prospect theory in econometrics, where individuals were more sensitive to losses (e.g., diabetes complications) than to equivalent gains.^54^ The logarithmic term reflected diminishing marginal sensitivity to change in risk as in equation 11:

(11) $$RA_{i,t}=\theta_{s^{\prime }}\cdot SI_{i,t-1}-\theta_{r^{\prime }}\cdot \log\left(1+RA_{i,t-1}\right)$$

where $SI_{i,t-1}$ is the severity index for diabetes progression in time *t*− 1 defined in equation 5. $RA_{i,t-1}$ is the previous risk aversion behavior in time *t*− 1 and $\theta_{s^{\prime }},\theta_{r^{\prime }}$ are weight parameters for severity and risk behavior.

#### Choice of service function

The choice of health care provider $CS_{i,t}$ was modeled as a utility maximization problem,^45^ where the agent selected a provider *j* that maximized their combined hedonic and normative utility as in equation 12:

(12) $$CS_{i,t}=\underset{\;j}{\arg\;\max}\left(\theta_{h}\cdot HG_{i,j,t}+\theta_{v}\cdot NG_{i,j,t}\right)$$

where $HG_{ij,t}$ is the hedonic goal related to the provider j (e.g., convenience, comfort) and $NG_{\mathrm{ij},t}$ is the normative goal, such as social pressure to visit a particular provider. $\theta_{o},\theta_{v}$ are weight parameters for hedonic and normative goals.

#### Hedonic function

The hedonic goal $HG_{i,j,t}$ represented the personal utility agent i derived from accessing health care provider j , focusing on travel distance and time. The agent experienced disutility from longer distances and travel times,^50^ which reduced the attractiveness of a health care provider as in equation 13.

(13) $$HG_{i,j,t}=1-\left(\theta_{d}\cdot \frac{\mathrm{DI}S_{i,j}}{\mathrm{DI}S_{\max}}+\theta_{t}\cdot \frac{TT_{i,j,t}}{TT_{\max}}\right)$$

where $DIS_{i,j}$ is the distance to the nearest health care facility and $\mathrm{DI}S_{\max}$ is the maximum possible distance, used for normalization. $TT_{i,j,t}$ is the travel time required for agent i to reach health care provider j , while TT is the maximum possible travel time within the region. $\theta_{d}$ is the weight parameter for the disutility associated with travel distance, reflecting how sensitive the agent was to travel distance, and $\theta_{t\;}$ is the weight parameter for the disutility associated with travel time, reflecting how sensitive the agent was to the time taken to reach the healthcare provider.

#### Normative function

The normative goal $NG_{i,j,t}$ captured the nonlinear effects of reputation and trust on agent i ’s evaluation of health care provider j . This formulation assumed that the importance of reputation and quality could grow or diminish at varying rates depending on their initial values as in equation 14.

(14) $$NG_{i,j,t}=\theta_{r}\cdot {\left(RP_{i,j,t-1}\right)}^{\alpha_{r}}+\theta_{q}\cdot {\left(TR_{i,j,t-1}\right)}^{\alpha_{q}}$$

Where $RP_{i,j,t-1}$ is the reputation score of health care provider *j*, reflecting community feedback and satisfaction at time *t* as in equation 3. $TR_{i,j,t-1}$ is the perceived trust in health care provider j at time *t* as in equation 1. $\theta_{r}$ is a weight parameter for reputation and $\theta_{q}$ is a weight parameter for perceived trust. $\alpha_{r},\alpha_{q}$ are parameters controlling the nonlinear effects of reputation and trust, where values greater than 1 reflect accelerating returns and values less than 1 reflect diminishing returns.

### Experimental Design and Scenario Analysis

Following guidance from the NSW Ministry of Health and EY Public Policy Group, we designed 3 critical experiments and 2 scenarios to address fundamental gaps in understanding health-seeking behavior through social-behavioral frameworks. Each analysis, which was replicated 100 times with 95% confidence intervals, targeted distinct yet interconnected aspects of health care decision making that had significant implications for public health policy and health care delivery optimization.

#### Experimental setting

The first experiment, focusing on TPB, addressed the crucial need to understand psychological barriers to health care access. The investigation, informed by prior findings,^55^ was particularly significant for identifying modifiable factors to improve early intervention rates. By examining how personal capability perceptions, motivations, and social pressures influenced health care decisions, this experiment aimed to inform targeted interventions that could overcome psychological barriers to seeking timely medical attention.

The second experiment, centered on HBM, tackled the critical challenge of understanding risk perception in health care decisions. Drawing on the existing studies,^56^ this experiment was essential for understanding how different risk attitudes influenced preventive health behaviors. Of particular interest was understanding how risk perception influenced the timing of health care seeking, which could inform strategies to promote earlier diagnosis and reduce health care costs.

The third experiment, integrating TPB and HBM, represented a novel approach to understanding the complex interplay of psychological and risk assessment factors in health-seeking behavior. This integration, supported by previous research,^57,58^ aimed to reveal how combined theoretical frameworks could better predict and influence health-seeking patterns, potentially leading to more effective public health strategies.

Through all the experiments, GFT provided a consistent framework for understanding health care provider selection, because it captured fundamental decision-making mechanisms that remained stable regardless of other behavioral influences.

#### Intervention assessment

By establishing a baseline theory within our experimental framework, we were able to identify which theoretical approach served best to guide our understanding of intervention impacts. Building on this foundation, we examined 2 scenarios to evaluate potential intervention strategies: increased awareness interventions and enhanced health care access programs. The increased awareness scenario simulated the implementation of targeted health education campaigns, incorporating modifications to agents’ attitude and social norm parameters (health literacy scores by +20% and social interactions parameters by +15%). The enhanced access scenario modeled improvements in health care infrastructure and service availability, adjusting PBC and gain function parameters (perceived barriers to care by −30% and distance to nearest care by −25%).

## Results

### Model Standard Tests: Sensitivity Analysis, Calibration, and Validation

**Figure 3:**
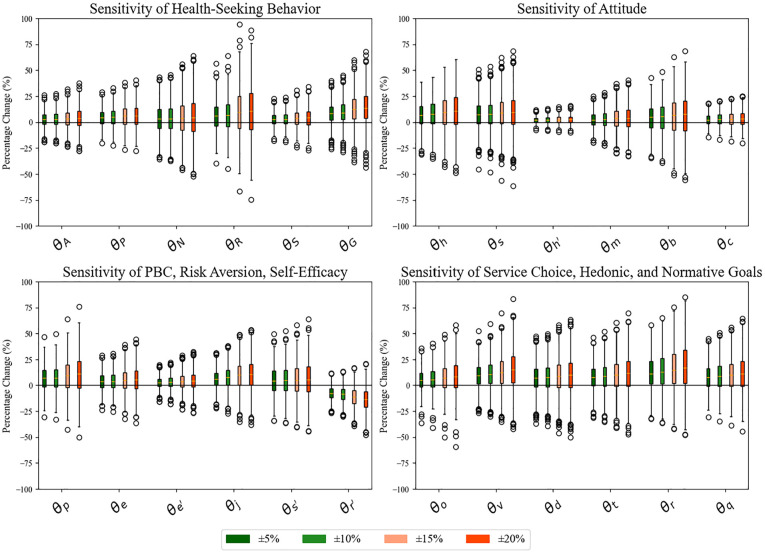
Model sensitivity analysis, showing the percentage change in outcomes for variations in θ .

To evaluate model robustness, we conducted a comprehensive sensitivity analysis of key control parameters as shown in Figure 3. Following the one-factor-at-a-time method,^59^ each parameter was systematically varied by ±20% across 1,000 simulation runs with different random seeds to account for stochastic effects. The analysis revealed robust model responses to parameter variations across different behavioral components.

When examining the weight parameters ( θ ), most components demonstrated moderate sensitivity within the ±20% parameter variation, with the model maintaining stability particularly in the ±5% to 10% range. Notably, health-seeking behavior showed heightened sensitivity to social norm parameters and risk aversion coefficients ( $\theta_{N},\theta_{R}$ ), suggesting these are critical determinants in the decision-making process. The attitude component displayed relatively symmetric responses to parameter variations, while PBC, self-efficacy, and risk aversion parameters exhibited more asymmetric effects, particularly in the negative direction. The service choice, hedonic, and normative goal parameters demonstrated different threshold effects, where small variations (±5%) produced minimal changes but larger variations (±15%–20%) led to substantial shifts in behavior patterns. This was particularly evident in the upper quartile of the response distribution. The nonlinear response pattern was aligned with established behavioral theories suggesting stepped rather than continuous changes in health-seeking decisions. The model robustness was particularly evident in the consistent central tendencies across multiple parameter variations, with most interquartile ranges remaining within ±25% of baseline values, indicating reliable predictive capability.

Model calibration involved iteratively adjusting weight parameters ( θ,λ,α,γ,δ ) until the simulated outputs matched confidential patient data across: the proportion of patients developing cardiovascular complications within 5 y of late diagnosis, the time between diagnosis and first hospital admission for diabetes-related complications, and the annual rate of preventable hospitalizations. The process involved running *t* tests between simulated and observed data, resulting in *P* values of 0.062, 0.073, and 0.124, respectively, for these respective metrics. While 2 of the 3 the *P* values were close to 0.05, the third was less so. However, the alignment was deemed sufficient for practical purposes in health care modeling, as it did not significantly affect decision-making outcomes or the clinical relevance of the results. These parameters were further fine-tuned using maximum likelihood estimation to minimize the differences, with particular attention to the temporal aspects of disease progression and health care utilization patterns. More information is available in Appendix A.2.

**Figure 4:**
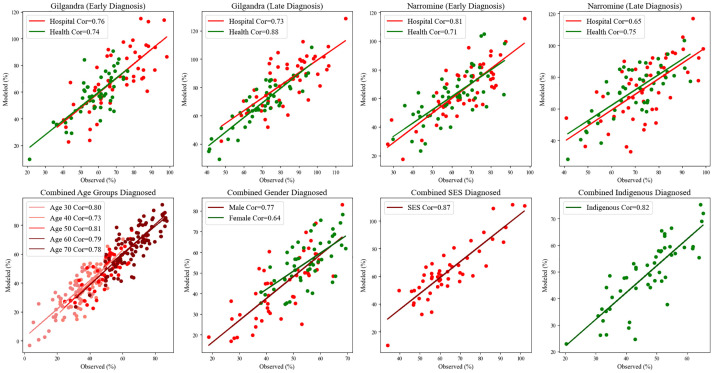
Scatter plots to compare the simulated and observed percentages for early and late diagnoses, as well as by demographic factors.

Model validation was conducted using 2 distinct datasets: primary health data from NSW diabetes patients (obtained through consultation with the Ministry of Health, subject to Data Protection Act 2018) and interpolated public data from AIHW. The validation results demonstrated robust model performance across different geographic and demographic contexts, with correlation coefficients ranging from 0.65 to 0.88, indicating strong predictive capability (refer to Figure 4). Notably, the model showed higher accuracy in early diagnosis scenarios (correlation coefficients: 0.74–0.81) compared with late diagnosis cases (correlation coefficients: 0.65–0.75), suggesting better predictive power for preventive healthcare behaviors.

Demographic-specific validation revealed particularly strong model performance across SES groups (correlation coefficient: 0.87) and Indigenous populations (correlation coefficient: 0.82), indicating the model’s effectiveness in capturing diverse population characteristics. Age-stratified analysis showed consistent performance across different age groups (correlation coefficients: 0.73–0.81), with slightly better prediction accuracy for age groups 30 and 50 (correlation coefficients: 0.80 and 0.81, respectively). Gender-based validation indicated stronger predictive power for male health-seeking behavior (correlation coefficient: 0.77) compared with female (correlation coefficient: 0.64), suggesting potential areas for model refinement in capturing gender-specific health-seeking patterns. The geographical comparison revealed that in Gilgandra, the model showed stronger prediction accuracy for hospital visits in early diagnosis scenarios (correlation coefficient: 0.76) compared with health center visits (correlation coefficient: 0.74), while Narromine displayed an inverse pattern with better prediction accuracy for health center utilization in late diagnosis cases (correlation coefficient: 0.75).

In addition to quantitative validation, qualitative validation was conducted through participatory workshops with stakeholders. These workshops allowed health care professionals and policy makers to examine and discuss different model functions, particularly those associated with early diagnosis, late-diagnosis rates, and high-risk demographics. Insights gathered from these discussions were instrumental in refining the model to align with industry practices and expert knowledge. Furthermore, the model was presented at 2 conferences (IEEE Transaction on Engineering Management and MODeling and SIMulation) and 2 industry meetings with key stakeholders to solicit additional feedback from a wider audience of professionals. This process of face validation through external feedback further strengthened the model reliability and alignment with current practices in diabetes care.

### Experiment Analysis: Diagnosis Time, Performance, Cost

#### Temporal patterns in diabetes diagnosis across health care settings

**Figure 5.**
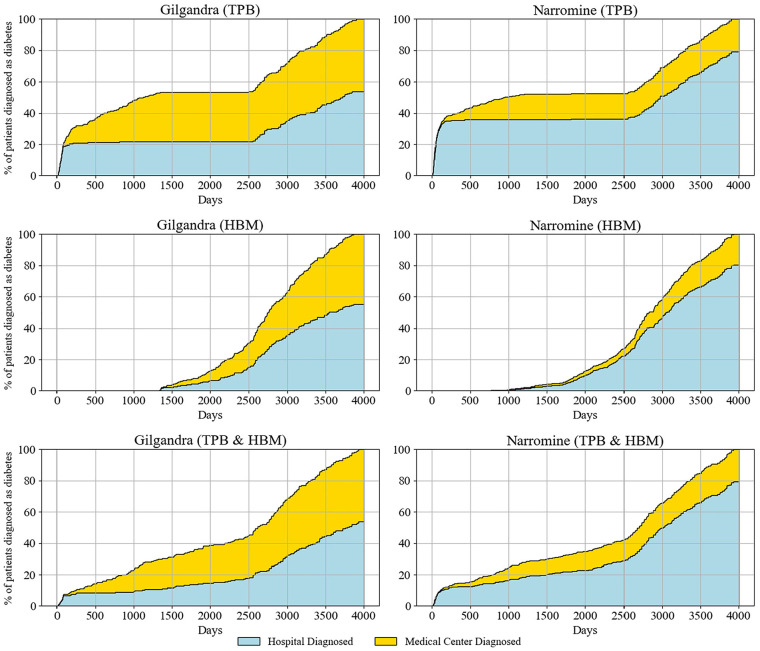
Percentage of patients diagnosed by medical centers and hospitals in 3 behavioral experiments in Gilgandra and Narromine.

The longitudinal analysis of diabetes diagnosis patterns identified distinct trends across health care settings, geographical regions, and theoretical frameworks (Figure 5). When examining the combined TPB and HBM scenarios, which provided the most comprehensive representation of health care–seeking behaviors, we observed notable differences between the 2 study locations. In Gilgandra, hospitals accounted for approximately 60% of diagnoses, with medical centers responsible for the remaining 40%. This ratio was maintained consistently over the 4,000-d observation period. In contrast, Narromine exhibited a more pronounced reliance on hospital-based diagnoses (80%) compared with medical centers (20%), potentially reflecting the community’s limited access to primary health care facilities.

The TPB scenarios demonstrated continuous diagnosis patterns, with steady increases in both hospital and medical center diagnoses over time. Particularly noteworthy is the acceleration in diagnosis rates observed after day 2,500 across all scenarios, potentially indicating increased health awareness. The isolated HBM scenarios unveiled a more distinctive temporal pattern characterized by a significant diagnostic gap extending for approximately 1,000 d, during which new diagnoses were minimal. Importantly, this gap was substantially reduced in the combined TPB-HBM model, suggesting that integrating both theoretical frameworks better captured the complexities of health care–seeking behaviors. The combined TPB-HBM framework provided the most realistic representation of diagnosis patterns, addressing limitations identified in previous single-framework studies.^60^ This integrated approach captured both planned and unplanned health care visits, with approximately 60% of diagnoses occurring through intentional appointments before day 3,000; this corroborated findings from O’Connor et al.^61^ and Fleig et al.^62^ The remaining 40% resulted from unplanned visits, highlighting the crucial role of maintaining robust emergency and walk-in services.

#### Temporal patterns in health care utilization and diagnostic performance

**Figure 6:**
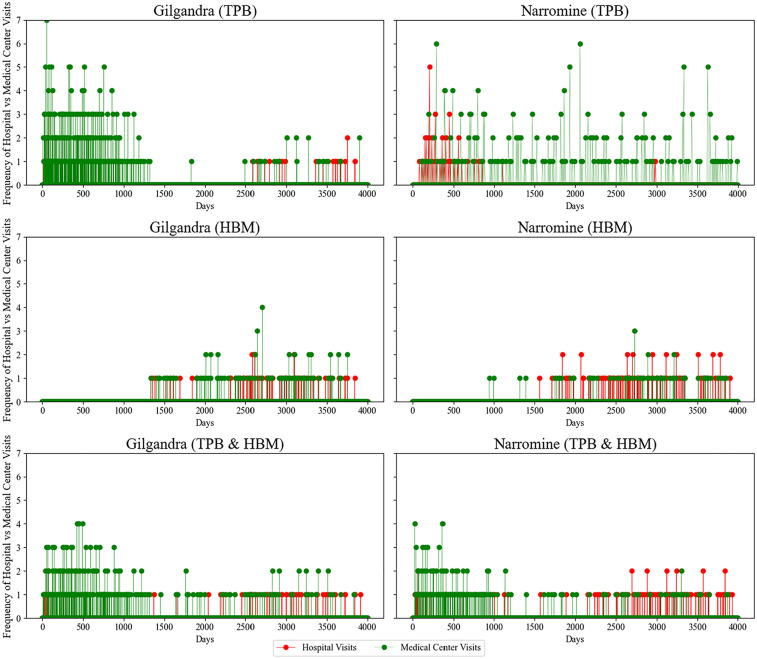
Health care visit frequency patterns in 3 behavioral experiments in Gilgandra and Narromine.

Figure 6 reveals several compelling temporal patterns across theoretical frameworks. In the TPB model, Gilgandra exhibited a distinctive early-stage clustering of medical center visits (days 0–1,000), with visit frequencies reaching up to 7 visits per year. This intense early utilization pattern differed from Narromine, where visits were more evenly distributed but less frequent (maximum 6 visits/y). This disparity suggested that TPB-driven behavior was more effective in communities with better health care accessibility. The HBM implementation demonstrated a markedly different pattern, characterized by delayed but more consistent health care engagement. Notably, both regions showed reduced early-stage visit frequency compared with TPB but maintained steady visitation patterns after 1,500 d. This suggests that health belief–driven behaviors may have led to more sustained, though less intensive, health care engagement. A particularly interesting finding was the emergence of coordinated hospital-medical center visit patterns in later stages (>,2500 d), indicating improved care coordination under HBM. The combined TPB-HBM framework yielded the most balanced utilization pattern. In Gilgandra, we observed that early engagement was maintained (although less intensely than TPB only) along with consistent long-term follow-up. Narromine’s pattern showed improved early engagement compared with HBM only, while maintaining the steady long-term pattern. This suggested that the combined model effectively balanced immediate health-seeking behavior with sustained engagement in line with our observation of the real-world pattern.

**Figure 7:**
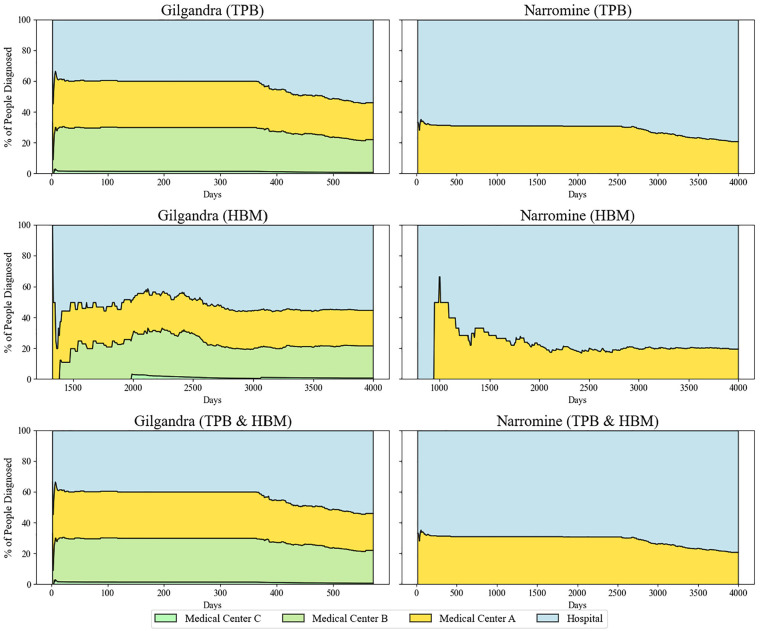
Diagnostic performance across health care settings in 3 behavioral experiments in Gilgandra and Narromine.

The diagnostic performance data identified striking differences in health care delivery effectiveness across frameworks and regions (Figure 7). Under TPB, Gilgandra demonstrated a 3-tier diagnostic distribution (approximately 30% medical center B, 30% medical center A, 40% hospital) that remained relatively stable until day 400. This stability suggested effective primary care capacity but also indicated potential system redundancy. Narromine’s TPB pattern showed a stark hospital dominance (>70%), highlighting infrastructure disparities between regions. The HBM framework produced an unexpected pattern in Gilgandra, with gradual increases in medical center A’s diagnostic share (reaching 35% by day 2,000) and corresponding decreases in hospital diagnoses. This suggests that health beliefs may drive patients toward primary care settings when available. Narromine’s HBM pattern showed minimal improvement in primary care utilization, reinforcing the impact of infrastructure limitations on behavior-based interventions. The combined TPB-HBM framework demonstrated superior diagnostic distribution, particularly in Gilgandra, maintaining primary care diagnosis rates greater than 50% throughout the observation period. This optimal distribution aligns with health care system design principles that favor primary care–led diagnosis. Notably, the combined model also showed the most stable long-term patterns, suggesting enhanced system sustainability.

#### Diagnosis cost for a health care system

**Figure 8:**
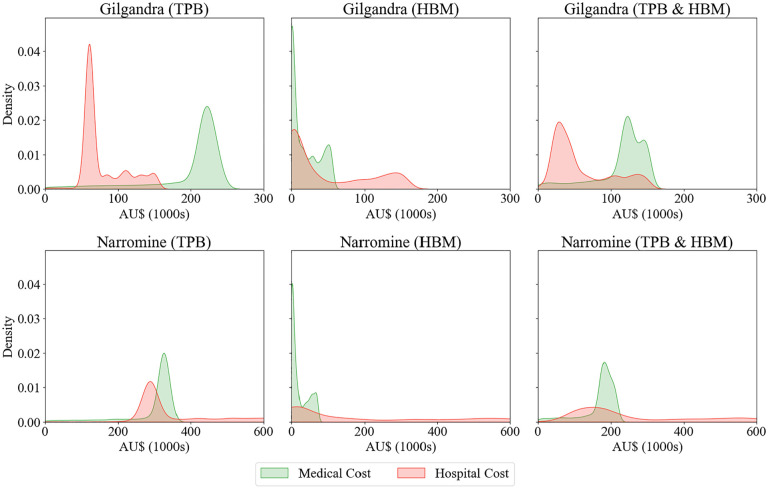
Kernel density estimates of medical and hospital costs across different behavioral experiments in Gilgandra and Narromine.

Figure 8 presents the estimated density distributions of medical and hospital costs under 3 behavioral scenarios. We approximated the medical center costs of diabetes screening at AU$100 per individual considering a diverse range of factors such as diagnostic tests, medical consultation fees, patient hospitalization costs, and associated administrative expenses. Sources for these components included national diabetes prevalence rate, ABS, and expert advice, all carefully calibrated to echo the specific circumstances of our study locations.

For Gilgandra, the TPB framework showed a bimodal distribution with sharp peaks in hospital costs of about AU$50,000 and medical costs of about AU$220,000. However, when examining the HBM scenario, we observed a dramatically different pattern with high-density early costs and a more gradual decline, particularly in hospital costs. The combined TPB and HBM scenario produced what appeared to be a more balanced distribution, with smoother peaks and a more realistic overlap between medical and hospital costs, suggesting a more accurate representation of real-world health care utilization patterns.

In Narromine, the cost distributions showed notably different characteristics. The TPB scenario demonstrated overlapping medical and hospital cost densities centered around AU$300,000, while the HBM scenario showed a pronounced early peak in medical costs with a rapid decline. The combined TPB and HBM scenario for Narromine revealed a distinctive pattern in which medical costs peaked at about AU$200,000, with hospital costs showing a broader, more diffuse distribution. This aligned with previous research suggesting that rural areas often experience different health care utilization patterns owing to accessibility factors.

When considering the total population of these regions and typical screening costs per individual, the density distributions of the TPB and HBM model suggested plausible aggregate costs for both medical centers and hospitals. This provided an important validation of the model’s outputs, because the total costs for Gilgandra at AU$250,000 and Narromine at AU$350,000 fell within expected ranges for population health screening programs in these rural regions (approximately AU$270,000 for Gilgandra and AU$380,000 for Narromine, based on ABS data and expert panel consensus). The higher medical center costs compared with hospital costs also reflected realistic health care expenditure patterns.

### Scenario Analysis: Increased Awareness versus Enhanced Primary Care Access

**Figure 9:**
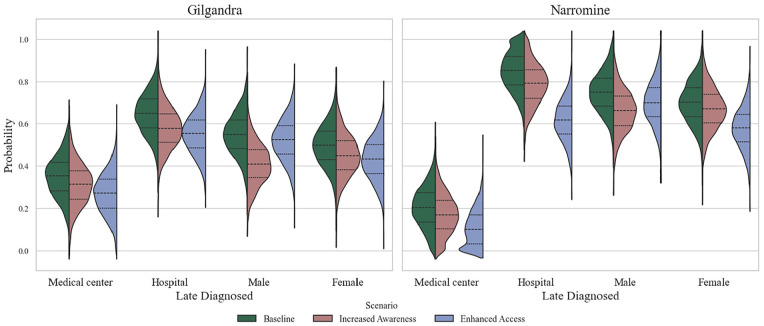
The distribution of late diagnosis probabilities under 2 scenarios: increased awareness and enhanced access, for both Gilgandra and Narromine.

Given that the multitheory framework demonstrated the best performance in our experimental analysis, we selected it as the baseline for further investigation. The scenario analysis in Figure 9 revealed distinct patterns in late diagnosis probabilities across health care settings and demographic groups in Gilgandra and Narromine, comparing baseline conditions with increased awareness and enhanced health care access interventions. This analysis examined late diagnosis probabilities across 3 key dimensions: health care facility type (hospitals versus medical centers), geographical location (Gilgandra versus Narromine), and gender distribution. In Gilgandra, the violin plots demonstrated a moderate but systematic reduction in late diagnosis probabilities across all categories following intervention implementation. Medical centers showed baseline probabilities centered around 0.35 to 0.40 (green distribution), which decreased to approximately 0.30 to 0.35 under increased awareness (red distribution) and further reduced to 0.25 to 0.30 with enhanced access (blue distribution).

Narromine presented markedly different baseline conditions and intervention responses. The baseline scenario revealed notably higher late diagnosis probabilities (0.80–1.0) across all categories compared with Gilgandra, with particularly elevated rates in hospital settings. Medical centers in Narromine showed dramatic improvements under enhanced access interventions, with probability distributions shifting from baseline peaks of about 0.85 to approximately 0.40 to 0.45. This represented one of the most substantial improvements observed across all scenarios. Hospital-based late diagnoses in Narromine demonstrated a distinctive nonlinear response pattern, in which enhanced access interventions produced a bimodal distribution (blue) centered around 0.50 and 0.70, suggesting complex underlying dynamics in health care–seeking behavior.

Gender-specific analysis revealed that in Gilgandra, males showed particularly strong responses to awareness interventions, with their late diagnosis probabilities decreasing from approximately 0.60 to 0.45, while females demonstrated more modest improvements from 0.55 to 0.45. However, in Narromine, females exhibited more pronounced responses to enhanced access interventions, with their probability distributions showing a more concentrated peak of about 0.50 to 0.55 compared with males’ broader distribution centered about 0.60 to 0.65. This gender disparity was particularly evident in the enhanced access scenario, in which the female distribution showed a more defined peak and narrower spread compared with the male distribution.

## Discussion

Our findings identified the complex relationships among the behavioral, geographical, and health system factors in shaping health care–seeking patterns in diabetes diagnosis. They addressed our 3 primary RQs while offering several insights that merit further discussion.

Regarding our first RQ about the effectiveness of integrated behavioral models, the results demonstrated that the integrated TPB-HBM-GFT framework consistently outperformed single-theory models in predicting diagnosis patterns and health care utilization (RQ-I). This superior performance manifested particularly in the elimination of the significant diagnostic gap (approximately 1,000 d) observed in the HBM-only scenarios, suggesting that the integration of planned behavior elements with health beliefs creates a more realistic representation of health care–seeking decisions. This finding challenges the traditional single-theory approaches that are prevalent in health behavior modeling.^63–65^ It supports the argument for more comprehensive theoretical frameworks that can capture both planned and reactive health-seeking behaviors.

Moreover, the temporal patterns revealed distinct regional variations in the effectiveness of behavioral frameworks. In Gilgandra, the TPB framework showed strong early-stage clustering of medical center visits (up to 7 visits/y), aligning with Conner and Armitage’s findings on intention-driven health care–seeking behavior.^41^ However, this pattern demonstrated a significant decline in the middle phase of diabetes, with a notable shift toward hospital visits in later stages, likely due to complications and comorbidities from disease progression. In contrast, Narromine exhibited more distributed but less frequent patterns. This unexpected disparity suggests that planned behavior-driven interventions may be more effective in communities with better health care accessibility, challenging the assumption that behavioral frameworks have uniform effects across different health care contexts. The emergence of coordinated hospital-medical center visit patterns in later stages (>2,500 d) under the HBM framework indicated that health belief–driven behaviors might promote better care coordination over time, also highlighted in Kishore et al.^66^

It should be noted that the framework effectiveness varied slightly between early and late diagnosis scenarios, with correlation coefficients ranging from 0.74 to 0.81 for early diagnosis compared with 0.65 to 0.75 for late diagnosis cases. This variation suggests that while the framework can improve overall predictive power, it may still have limitations in capturing the complexity of delayed health care–seeking behaviors.

The second RQ (RQ-II), focusing on health care costs and diagnostic performance, revealed several counterintuitive patterns. Despite higher overall costs, hospital-based diagnoses did not necessarily lead to better health outcomes. This was particularly evident in Narromine, where more than 70% of diagnoses occurred in hospitals under the TPB framework. The high reliance on hospital services suggested inefficiencies in the health care system, because many cases that could have been effectively managed through earlier and less costly medical center interventions ended up being treated in hospitals, driving up expenses without necessarily improving patient outcomes. The cost density distributions showed bimodal patterns in Gilgandra under the TPB framework, with peaks around AU$50,000 for hospital costs and AU$220,000 for medical costs, indicating that behavioral factors influenced the timing and the cost trajectory of diabetes diagnosis. This finding challenges conventional cost-effectiveness models that often assume linear relationships between health care access and costs.^47^ These nonlinear patterns have crucial implications for policy design, suggesting that straightforward cost reduction strategies may yield unexpected results.

Perhaps most notably, the analysis of diagnostic performance revealed a 3-tier distribution in Gilgandra (30% medical center B, 30% medical center A, 40% hospital) that remained stable until day 400. This suggested an unexpected level of system resilience. The stability, however, came with potential redundancy costs, highlighting the complex tradeoffs between system reliability and efficiency. The gradual increase in medical center A’s diagnostic share under the HBM framework (reaching 35% by day 2,000) suggests that health beliefs may drive patients toward primary care settings when available, a finding that has significant implications for health care resource allocation.

Addressing our third RQ (RQ-III) on theory-based interventions, the scenario analysis revealed surprising gender-specific responses to interventions. In Gilgandra, males showed stronger responses to awareness interventions (probability decreased from 0.60 to 0.45) compared with females (0.55 to 0.45), while in Narromine, females demonstrated more pronounced responses to enhanced access interventions. This gender disparity in intervention effectiveness challenges the one-size-fits-all approach to health care interventions and suggests the need for more tailored, gender-specific strategies in diabetes prevention programs.

These results have significant implications for both theory and practice. On the theoretical front, they underscore the value of multitheory frameworks and ABM simulation in health behavior research, particularly for complex chronic conditions such as diabetes. While traditional empirical studies are constrained by available data and offer only static snapshots, ABM can simulate dynamic responses even where data are scarce by drawing on theoretical principles. From a practical standpoint, our results suggest that health care interventions should be customized to specific regional and demographic contexts rather than applied uniformly across different settings. Importantly, we found that simply improving health care access is not enough—effective interventions must address both structural obstacles (like transportation and cost) and behavioral barriers (such as health beliefs and risk awareness) to achieve earlier diagnosis.

This study directly contributes to the Australian National Diabetes Strategy 2021–2030,^67^ particularly goal 7 to strengthen prevention and care through research, evidence, and data. Goal 7 highlights 2 areas of action: to develop a national research agenda and improve and expand data linkage and facilitate ease of access. The strategy mentions the need for improved technologies for informing health policy decisions and for targeted decision making. This study directly contributes to the strategy by showcasing a collaborative approach for creating a decision support tool to enable policy makers and health care providers to assess and manage targeted interventions for diabetes control and early diagnosis of the disease. Furthermore, the tool offers the capacity to enable optimization and timely access to resources. The tool would also enable an evidence base for planning for future health policy and services for effective health care delivery and to provide adequate support to patients at the right time. The tool has been developed such that it can be adapted and applied for modeling diseases other than diabetes, such as cardiovascular diseases that are a leading cause of death in Australia.^68^

## Conclusion

This study advances the scholarly discourse on health care–seeking behavior in several significant dimensions. By synthesizing multiple theoretical frameworks within an ABM approach, we transcended the limitations of conventional single-theory paradigms in understanding diabetes diagnosis patterns across rural Australian settings. The model’s sophisticated integration of behavioral determinants revealed previously unexamined heterogeneity in health care utilization across geographic and demographic strata. The granular analysis of regional variations demonstrated how spatially differentiated approaches to diabetes screening and diagnosis could optimize resource allocation while addressing community-specific barriers to care. These insights hold particular salience for health system stakeholders who are seeking evidence-based strategies to mitigate delayed diagnosis in resource-constrained environments.

Several limitations of this study provide avenues for refinement. First, the model parameters, while derived from a combination of empirical data and expert insights, were limited by the availability and specificity of rural health care data in Australia. The study used synthesized estimates for model initialization, meaning that some parameters may not fully reflect local variations in health-seeking behaviors. Although sensitivity analyses were conducted to test parameter robustness, access to finer-grained real-time data could enhance model accuracy. Another limitation was that the ABM focused specifically on rural contexts with limited generalizability to urban areas, where health care infrastructure and socioeconomic factors may differ significantly. In addition, this model did not fully incorporate macroeconomic or policy changes that can significantly affect health care access, which could be particularly relevant in the wake of health policy reforms or economic shifts.

Future work should consider several avenues for advancing the framework and application of this model. First, incorporating real-time data, such as electronic health records, could refine the model parameters and improve the accuracy of predictions in the context of evolving health care demands. Extending the ABM to urban settings and examining other chronic diseases, such as cardiovascular disease, could also test the versatility of the theoretical framework across diverse health contexts. Exploring digital health literacy and telemedicine access, especially for remote communities, may further enhance the model’s scope and relevance, providing insights into modern, technology-driven solutions for health care access. Another promising direction for future research lies in the integration of policy and economic factors into the model to assess the broader impact of health care policies on health-seeking behaviors. For instance, the inclusion of simulated scenarios that incorporate economic factors such as subsidies or insurance coverage changes could offer policy makers insights into the effectiveness of targeted health care funding. This study provides a strong foundation for advancing health care behavior modeling in chronic disease contexts, supporting the development of interventions that are both behaviorally informed and contextually sensitive. Interdisciplinary research focusing on integrating supply chain modeling techniques, such as input–output modeling and life-cycle assessment, with agent-based modeling can enable the integrated assessment of health outcomes and the impact of those outcomes on the environment, society, and economy. Such an integrated approach has the potential to codevelop environmentally and socially sustainable health interventions for diagnosing and treating diseases.

## Figures and Tables

**Figure A.1:**
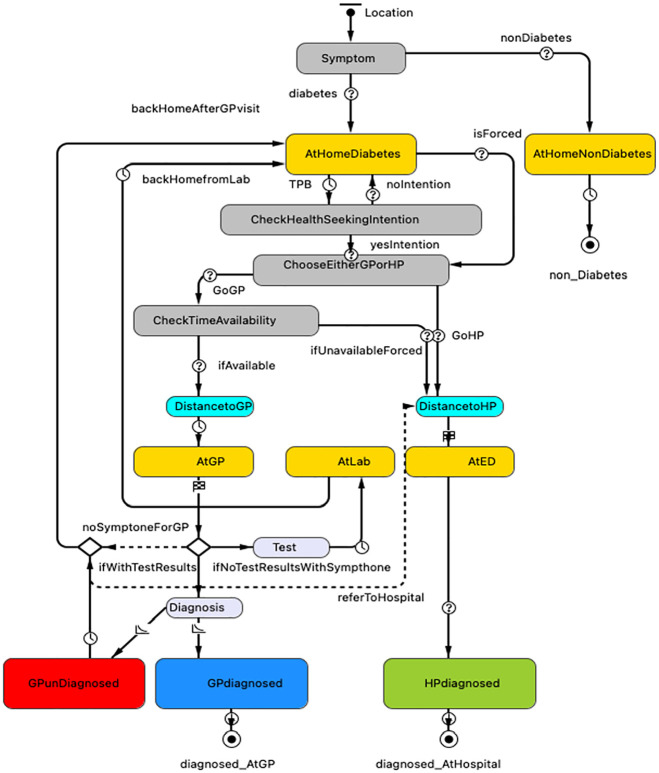
The state transition diagram for the patient agent. Patients can be at an undiagnosed state at home, diagnosed state at hospital, and both diagnosed and undiagnosed at medical centers (indicated as GP).

## Appendix A

### A.1. Patient Decision-Making in Health Care Systems

The state transition diagram in Figure A.1 illustrates the journey of a patient agent within the health care system. Each patient agent started in the “undiagnosed” state at home, representing the initial stage at which diabetes symptoms might or might not have been present. When symptoms arose, the patient assessed their intention to seek medical care, which determined whether they transitioned to the “seeking a health care provider” state. Patients could receive diagnoses at either medical health centers (indicated as general practitioners [GPs]) or hospital emergency departments, leading to a “diagnosed” state. The distinction between being diagnosed at a hospital versus a medical health center enabled the modeling of health care pathways, incorporating factors such as diagnostic accuracy, referral processes, and urgency of treatment. This diagram displayed how agents transitioned between undiagnosed and diagnosed states, driven by symptoms, diagnostic success, and referral mechanisms.

Table A.1 outlines the decision flow for patient and health care provider agents, highlighting the interactive, sequential actions within the model. Patient agents began at home and first checked for diabetes symptoms. If symptoms were present, patients assessed their intention to seek care, which was influenced by factors such as perceived barriers and social norms. Based on this intention, patients selected a health care provider, either a hospital or a medical center, where different diagnostic processes were modeled. Medical centers could refer patients to hospitals in urgent cases, capturing real-world dynamics of health care decision making and referral pathways. For health care providers, the table details the setup processes, such as defining service costs, operating hours, and diagnostic success rates, which align with the standards of New South Wales Health and the Victorian Department of Health. This action flow modeled patient and provider decisions with accuracy, enabling detailed representations of patient journeys and the impact of provider characteristics on health care–seeking behaviors.

**Table A.1:** Flow of Actions for Patient and Health Care Provider Agents.

Patient Agent	Hospital and Medical Health Center Agent
1. START at home location in a GIS-based environment. 2. IF no diabetes symptoms, STAY at home. END. 3. IF diabetes symptoms, CHECK health-seeking intention. - *IF has no intention to seek medical care, STAY at home. END.* - *IF has an intention to seek medical care, PROCEED to step 4.* 4. CHOOSE health service provider (hospital or medical health center). - *IF hospital is selected, PROCEED to step 5.* - *IF medical health center is selected, PROCEED to step 6.* 5. GO to hospital emergency department, GET diagnosed immediately. END. 6. VISIT medical health center. - *IF medical health center diagnosed, END.* - *IF not diagnosed or not referred for medical test by medical health center, RETURN home and follow-up.* - *IF medical health center failed diagnosis multiple times, VISIT hospital for further health advice.* - *IF immediate treatment needed, medical health center REFER to hospital immediately. END.*	1. SET cost of health service, working hours, and diagnosis success rates. 2. INCLUDE operating hours of medical health centers as per NSW Ministry of Health’s advice. 3. INCORPORATE factors such as cost, timing, and regularity of treatment as per the Victorian Government Department of Health’s confirmation. 4. USE this information to influence patients’ decisions about where to obtain service. END.

### Appendix A.2. Model Calibration

Table A.2 presents an exhaustive list of control variables and weight parameters used for calibrating patient behaviors, health care interactions, and system dynamics within the model. Parameters such as α , γ , and δ controlled learning dynamics, adjusting trust based on positive or negative diagnostic outcomes. Social factors, including frequency of interactions ( $\theta_{n}$ ) and normative influence ( $\theta_{v}$ ), allowed the model to capture variability in the effects of social networks on health-seeking behavior among different individuals. Parameters representing PBC, attitude, and self-efficacy reflected psychological factors that influence health decisions, while service choice factors such as travel time and distance ( $\theta_{d}$ , $\theta_{t}$ ) accounted logistical considerations. Suggested values and distributions for each variable supported the model calibration, enabling the simulation to accurately reflect population heterogeneity and real-world healthcare patterns. Each value was grounded in empirical or assumed ranges, providing flexibility during calibration while ensuring model stability.

**Table A.2:** Control Variables and their Values for Model Calibration.

Control Variable	Notation	Description	Suggested Value/Distribution
Learning			
Persistence of past trust	α	Captures continuity of trust from previous periods	Normal distribution, μ=0.7 , σ=0.1
Trust increase for success	γ	Adjusts increase in trust for successful diagnoses	Uniform distribution [0.3, 0.7]
Penalty for failed diagnosis	δ	Scales trust reduction when a diagnosis fails	Fixed at 0.05
Reputation			
Decay parameter for reputation	λ	Weights recent feedback in reputation	Uniform distribution [0.6, 0.9]
Social influence			
Time consistency window	Δt	Defines period for evaluating consistent behavior	Fixed at 5 time units
Frequency of interactions	$\theta_{n}$	Weight for frequency effect on social influence	Uniform distribution [0.4, 0.8]
PBC			
Impact of perceived barriers	$\theta_{p}$	Weight for external barriers on PBC	Normal distribution, μ=0.6 , σ=0.1
Impact of self-efficacy on PBC	$\theta_{e}$	Weight for self-efficacy in PBC	Uniform distribution [0.4, 0.7]
Attitude			
Weight for health status	$\theta_{h}$	Weight on health status in attitude	Fixed at 0.5
Weight for socioeconomic status	$\theta_{s}$	Weight on SES in attitude	Uniform distribution [0.3, 0.6]
Weight for health literacy	$\theta_{h^{\prime }}$	Weight on health literacy in attitude	Uniform distribution [0.3, 0.6]
Weight for mental distress	$\theta_{m}$	Weight on mental health in attitude	Normal distribution, μ=0.5 , σ=0.1
Weight for health behaviors	$\theta_{b}$	Weight on healthy lifestyle in attitude	Uniform distribution [0.4, 0.7]
Weight for cultural health beliefs	$\theta_{c}$	Weight on cultural health beliefs in attitude	Fixed at 0.3
Self-efficacy			
Previous self-efficacy weight	$\theta_{e^{\prime }}$	Weight on past self-efficacy	Normal distribution, μ=0.6 , σ=0.1
Impact of health status on self-efficacy	$\theta_{j}$	Weight on health status in self-efficacy	Uniform distribution [0.4, 0.6]
Risk aversion			
Severity weight	$\theta_{s^{\prime }}$	Weight on diabetes severity in risk aversion	Uniform distribution [0.4, 0.6]
Risk behavior weight	$\theta_{r^{\prime }}$	Weight on previous risk behavior	Uniform distribution [0.3, 0.5]
Choice of service			
Weight for hedonic goal	$\theta_{h}$	Weight for convenience in provider choice	Uniform distribution [0.2, 0.5]
Weight for normative goal	$\theta_{v}$	Weight for social influence in provider choice	Uniform distribution [0.4, 0.7]
Influence of distance	$\theta_{d}$	Weight parameter for disutility from travel distance	Fixed at 0.3 or Uniform distribution [0.3, 0.5]
Influence of travel time	$\theta_{t}$	Weight parameter for disutility from travel time	Normal distribution, μ=0.5 , σ=0.1
Normative evaluation			
Impact of reputation on normative goal	$\theta_{r}$	Importance of reputation in normative evaluation	Uniform distribution [0.5, 0.9]
Impact of trust on normative goal	$\theta_{q}$	Importance of trust in normative evaluation	Uniform distribution [0.5, 0.8]
Nonlinearity in reputation	$\alpha_{r}$	Controls accelerating/diminishing returns of reputation	Fixed at 1.2
Nonlinearity in trust	$\alpha_{q}$	Controls accelerating/diminishing returns of trust	Fixed at 1.0

## References

[1] World Health Organization. Diabetes facts. 2018. Available from: https://www.who.int/news-room/fact-sheets/detail/diabetes

[2] GBD 2021 Diabetes Collaborators. Global, regional, and national burden of diabetes from 1990 to 2021, with projections of prevalence to 2050: a systematic analysis for the global burden of disease study 2021. Lancet. 2023;402(10397):203–234. 37356446 10.1016/S0140-6736(23)01301-6 PMC10364581

[3] Diabetes Australia. Diabetes in Australia, 2024. Available from: https://www.diabetesaustralia.com.au/about-diabetes/diabetes-in-australia/. [Accessed 29 October, 2024].

[4] Valentine NA Alhawassi TM Roberts GW Vora PP Stranks SN Doogue MP . Detecting undiagnosed diabetes using glycated haemoglobin: an automated screening test in hospitalised patients. Med J Aust. 2011;194(4):160–4. 10.5694/j.1326-5377.2011.tb02954.x 21401454

[5] Singh BM Jackson DM Wills R Davies J Wise PH . Delayed diagnosis in non-insulin dependent diabetes mellitus. BMJ. 1992;304(6835):1154. 1392794 10.1136/bmj.304.6835.1154 PMC1882076

[6] UK Prospective Diabetes Study Group. Effect of intensive blood-glucose control with metformin on complications in overweight patients with type 2 diabetes (UKPDS 34). Lancet. 1998;352(9131):854–65. 9742977

[7] Jackson DM Wills R Davies J Meadows K Singh BM Wise PH . Public awareness of the symptoms of diabetes mellitus. Diabet Med. 1991;8(10):971–2. 10.1111/j.1464-5491.1991.tb01540.x 1838052

[8] Bellou V Belbasis L Tzoulaki I Evangelou E . Risk factors for type 2 diabetes mellitus: an exposure-wide umbrella review of meta-analyses. PLoS One. 2018;13(3):e0194127. 10.1371/journal.pone.0194127 PMC5860745 29558518

[9] Janz NK Becker MH . The health belief model: a decade later. Health Educ Q. 1984;11(1):1–47. 6392204 10.1177/109019818401100101

[10] Bandura A . Social cognitive theory of self-regulation. Organ Behav Hum Decis Process. 1991;50(2):248–87.

[11] Rosenstock IM . Historical origins of the health belief model. Health Educ Monogr. 1974;2(4):328–35. 10.1177/109019817800600406 299611

[12] Schulte PA Wagner GR Ostry A , et al. Work, obesity, and occupational safety and health. Am J Public Health. 2007;97(3):428–36. 10.2105/AJPH.2006.086900 PMC1805035 17267711

[13] Prochaska JO DiClemente CC . Transtheoretical therapy: toward a more integrative model of change. Psychother: Theory Res Pract. 1982;19(3):276.

[14] Rogers RW . A protection motivation theory of fear appeals and attitude change. J Psychol. 1975;91(1):93–114. 28136248 10.1080/00223980.1975.9915803

[15] Adler NE Newman K . Socioeconomic disparities in health: pathways and policies. Health Aff. 2002;21(2):60–76. 10.1377/hlthaff.21.2.60 11900187

[16] Ajzen I . The theory of planned behavior. Organ Behav Hum Decis Process. 1991;50(2):179–211.

[17] Berkman ND Sheridan SL Donahue KE , et al. Health literacy interventions and outcomes: an updated systematic review. Evid Rep Technol Assess. 2011;199:1–941. PMC4781058 23126607

[18] Lindenberg S Steg L . Goal-framing theory and norm-guided environmental behavior. In: van Trijp HCM , ed. Encouraging Sustainable Behavior: Psychology and the Environment. New York: Psychology Press; 2013. p 37–54.

[19] Alibrahim A Wu S . Modelling competition in health care markets as a complex adaptive system: an agent-based framework. Health Syst. 2020;9(3):212–25. 10.1080/20476965.2019.1569480 PMC7476531 32939260

[20] Chao D Hashimoto H Kondo N . Dynamic impact of social stratification and social influence on smoking prevalence by gender: an agent-based model. Soc Sci Med. 2015;147:280–7. 10.1016/j.socscimed.2015.08.041 26610078

[21] Nunner H Buskens V Teslya A Kretzschmar M . Health behavior homophily can mitigate the spread of infectious diseases in small-world networks. Soc Sci Med. 2022;312:115350. 36183539 10.1016/j.socscimed.2022.115350

[22] Marshall BDL Galea S . Formalizing the role of agent-based modeling in causal inference and epidemiology. Am J Epidemiol. 2015;181(2):92–9. 10.1093/aje/kwu274 PMC4351348 25480821

[23] Silverman BG Hanrahan N Bharathy G Gordon K Johnson D . A systems approach to healthcare: agent-based modeling, community mental health, and population well-being. Artif Intell Med. 2015;63(2):61–71. 25801593 10.1016/j.artmed.2014.08.006

[24] Tracy M Cerdá M Keyes KM . Agent-based modeling in public health: current applications and future directions. Annu Rev Public Health. 2018;39:77–94. 29328870 10.1146/annurev-publhealth-040617-014317 PMC5937544

[25] Karimi E Schmitt K Akgunduz A . Effect of individual protective behaviors on influenza transmission: an agent-based model. Health Care Manag Sci. 2015;18(3):318–33. 10.1007/s10729-014-9310-2 25578039

[26] Wood RM Pratt AC Murch BJ , et al. Establishing an seir-based framework for local modelling of covid-19 infections, hospitalisations and deaths. Health Syst. 2021;10(4):337–47. 10.1080/20476965.2021.1973348 PMC8567954 34745593

[27] Wai B Vasarhelyi K Rutherford AR , et al. A qualitative model of the HIV care continuum in Vancouver, Canada. Health Syst. 2022;11(2):84–97. 10.1080/20476965.2021.1906762 PMC9154767 35655610

[28] Alibrahim A Wu S . An agent-based simulation model of patient choice of health care providers in accountable care organizations. Health Care Manag Sci. 2018;21(1):131–43. 10.1007/s10729-016-9383-1 27704322

[29] Corsini RR Costa A Fichera S Pluchino A . A configurable computer simulation model for reducing patient waiting time in oncology departments. Health Syst. 2023;12(2):208–22. 10.1080/20476965.2022.2030655 PMC10208172 37234470

[30] Isern D Sánchez D Moreno A . Agents applied in health care: a review. Int J Med Inform. 2010;79(3):145–66. 10.1016/j.ijmedinf.2010.01.003 20129820

[31] Australian Institute of Health and Welfare. Diabetes: Australian facts. 2024. Available from: https://www.aihw.gov.au/reports/diabetes/diabetes/contents/how-common-is-diabetes/type-2-diabetes. [Accessed 24 October, 2024].

[32] Chen TH-H Yen M-F Tung T-H . A computer simulation model for cost-effectiveness analysis of mass screening for type 2 diabetes mellitus. Diabetes Res Clin Pract. 2001;54:37–42. 10.1016/s0168-8227(01)00307-2 11580967

[33] Zhou H Isaman DJM Messinger S , et al. A computer simulation model of diabetes progression, quality of life, and cost. Diabetes Care. 2005;28(12):2856–63. 10.2337/diacare.28.12.2856 16306545

[34] Borracci RA Giorgi MA . Agent-based computational models to explore diffusion of medical innovations among cardiologists. Int J Med Inform. 2018;112:158–65. 10.1016/j.ijmedinf.2018.02.008 29500015

[35] Grimm V Berger U Bastiansen F , et al. A standard protocol for describing individual-based and agent-based models. Ecol Modell. 2006;198(1–2):115–26.

[36] Glanz K Rimer BK Viswanath K . Health Behavior: Theory, Research, and Practice. San Francisco (CA): John Wiley & Sons; 2015.

[37] Compton MT Esterberg ML . Treatment delay in first-episode nonaffective psychosis: a pilot study with African American family members and the theory of planned behavior. Compr Psychiatry. 2005;46(4):291–5. 10.1016/j.comppsych.2004.10.006 16175761

[38] Adams C Gringart E Strobel N Masterman P . Help-seeking for mental health problems among older adults with chronic disease: an application of the theory of planned behaviour. Aust J Psychol. 2021;73(4):426–37.

[39] Rich A Brandes K Mullan B Hagger MS . Theory of planned behavior and adherence in chronic illness: a meta-analysis. J Behav Med. 2015;38:673–88. 10.1007/s10865-015-9644-3 25994095

[40] Lee LT Bowen PG Mosley MK Turner CC . Theory of planned behavior: social support and diabetes self-management. J Nurse Pract. 2017;13(4):265–70.

[41] Conner M Armitage CJ . Extending the theory of planned behavior: a review and avenues for further research. J Appl Soc Psychol. 1998;28(15):1429–64.

[42] Janz N Champion V Strecher V . The Health Belief Model. San Francisco (CA): Jossey-Bass; 2002.

[43] Daatland SO . Marital history and intergenerational solidarity: the impact of divorce and unmarried cohabitation. J Soc Issues. 2007;63(4):809–825.

[44] Stewart N Reimers S Harris AJL . On the origin of utility, weighting, and discounting functions: how they get their shapes and how to change their shapes. Manag Sci. 2015;61(3):687–705.

[45] Fishburn PC . Utility theory. Manag Sci. 1968;14(5):335–78.

[46] Hill-Briggs F Adler NE Berkowitz SA , et al. Social determinants of health and diabetes: a scientific review. Diabetes Care. 2021;44(1):258–79. 10.2337/dci20-0053 PMC7783927 33139407

[47] Kuske S Schiereck T Grobosch S , et al. Diabetes-related information-seeking behaviour: a systematic review. Syst Rev. 2017;6(1):212. 29065919 10.1186/s13643-017-0602-8 PMC5655894

[48] Ng BP LaManna JB Towne SD Jr Peach BC He Q Park C . Factors associated with avoiding health care among community-dwelling medicare beneficiaries with type 2 diabetes. Prev Chronic Dis. 2020;17:200148. 10.5888/pcd17.200148 PMC7587306 33059795

[49] Siegel KR Bullard KM Imperatore G , et al. Prevalence of major behavioral risk factors for type 2 diabetes. Diabetes Care. 2018;41(5):1032–9. 10.2337/dc17-1775 PMC6436527 29500166

[50] Du QH Zhang ZC Yang Y Luo XX Liu L Jia HH . How health seeking behavior develops in patients with type 2 diabetes: a qualitative study based on health belief model in China. Front Public Health. 2024;12:1414903. 39045167 10.3389/fpubh.2024.1414903 PMC11263333

[51] Reeves D Woodham AA French D , et al. The influence of demographic, health and psychosocial factors on patient uptake of the english nhs diabetes prevention programme. BMC Health Serv Res. 2023;23(1):352. 37041541 10.1186/s12913-023-09195-z PMC10091609

[52] Steinhaeuser K Chawla NV . A network-based approach to understanding and predicting diseases. In: Young MJ Salerno J Liu H , eds. Social Computing and Behavioral Modeling. New York: Springer; 2009. p 1–8.

[53] Silva-Tinoco R Cuatecontzi-Xochitiotzi T De la Torre-Saldaña V , et al. Influence of social determinants, diabetes knowledge, health behaviors, and glycemic control in type 2 diabetes: an analysis from real-world evidence. BMC Endocr Disord. 2020;20(1):130. 32843004 10.1186/s12902-020-00604-6 PMC7449009

[54] Schmidt U Zank H . Risk aversion in cumulative prospect theory. Manag Sci. 2008;54(1):208–16.

[55] Blue CL . Does the theory of planned behavior identify diabetes-related cognitions for intention to be physically active and eat a healthy diet? Public Health Nurs. 2007;24(2):141–50. 10.1111/j.1525-1446.2007.00618.x 17319886

[56] Charron-Prochownik D Sereika SM Becker D , et al. Reproductive health beliefs and behaviors in teens with diabetes: application of the expanded health belief model. Pediatr Diabetes. 2001;2(1):30–9. 10.1046/j.1399-543x.2001.00000.x 15016208

[57] Taghikhah F Voinov A Shukla N Filatova T . Exploring consumer behavior and policy options in organic food adoption: insights from the australian wine sector. Environ Sci Policy. 2020;109:116–24.

[58] Taghikhah F Filatova T Voinov A . Where does theory have it right? A comparison of theory-driven and empirical agent based models. J Artif Soc Soc Simul. 2021;24(2):12.

[59] Czitrom V . One-factor-at-a-time versus designed experiments. Am Stat. 1999;53(2):126–31.

[60] Nigg CR Allegrante JP Ory M . Theory-comparison and multiple-behavior research: common themes advancing health behavior research. Health Educ Res. 2002;17(5):670–9. 10.1093/her/17.5.670 12408211

[61] O’Connor PJ Gregg E Rush WA Cherney LM Stiffman MN Engelgau MM . Diabetes: how are we diagnosing and initially managing it? Ann Fam Med. 2006;4(1):15–22. 16449392 10.1370/afm.419 PMC1466993

[62] Fleig L Ngo J Roman B , et al. Beyond single behaviour theory: adding cross-behaviour cognitions to the health action process approach. Br J Health Psychol. 2015;20(4):824–41. 10.1111/bjhp.12144 26112344

[63] Getahun T Kaba M Derseh BT . Intention to screen for cervical cancer in debre Berhan town, Amhara regional state, Ethiopia: application of theory of planned behavior. J Cancer Epidemiol. 2020; 2020:5045671. 10.1155/2020/3024578 PMC7106926 32256590

[64] Bohon LM Cotter KA Kravitz RL Cello PC Jr Fernandez y Garcia E . The theory of planned behavior as it predicts potential intention to seek mental health services for depression among college students. J Am Coll Health. 2016;64(8):593–603. 27386898 10.1080/07448481.2016.1207646 PMC5181847

[65] Johnson CE Mues KE Mayne SL Kiblawi AN . Cervical cancer screening among immigrants and ethnic minorities: a systematic review using the health belief model. J Low Genit Tract Dis. 2008;12(3):232–41. 10.1097/LGT.0b013e31815d8d88 18596467

[66] Kishore J Kohli C Gupta N Kumar N Sharma P . Awareness, practices and treatment seeking behavior of type 2 diabetes mellitus patients in Delhi. Ann Med Health Sci Res. 2015;5(4):266–73. 10.4103/2141-9248.160184 PMC4512119 26229715

[67] Commonwealth of Australia. Australian National Diabetes Strategy 2021-2030. Canberra: Australian Government; 2021.

[68] Australian Bureau of Statistics. Causes of death, Australia, 2023. Available from: https://www.abs.gov.au/statistics/health/causes-death/causes-death-australia/2023. [Accessed 6 December, 2024].

